# Requisitioned academic time: A contradictory deconstruction of Chinese university faculty’s work priorities under the “Double First-Class” initiative

**DOI:** 10.1371/journal.pone.0356772

**Published:** 2026-09-29

**Authors:** Mengyun Fu, Jiajin Wu

**Affiliations:** Chinese Language and Cultural College, Huaqiao University, Xiamen, Fujian, People’s Republic of China; Islamic Azad University Mashhad Branch, IRAN, ISLAMIC REPUBLIC OF

## Abstract

This study examines the academic career development context in China through a temporal lens. Based on questionnaire data collected from university faculty across 12 Chinese provinces and municipalities, the analysis reveals three core findings. First, undergraduate teaching constitutes a compulsory and high-priority time block for Chinese university academics. Second, while most faculty aspire to prioritize academic writing, administrative tasks consistently take precedence over research in practice—a pattern rooted in the dominance of administrative power over academic authority in higher education institutions. Third, faculty’s expected priority for research time significantly outweighs its actual operational priority in daily work. Accordingly, this study proposes a tri-level governance framework to support the sustainable development of China’s academic profession.

## Introduction

In November 2015, the State Council of the People’s Republic of China issued the “Overall Plan for Coordinately Advancing the Construction of World First-class Universities and First-class Disciplines” to cultivate world-class universities and disciplines as a strategic measure to build China into a global powerhouse in higher education. This initiative is commonly referred to as the “Double First-Class” Initiative. Nowadays, with the advancement of modern information technology, the popularization of the internet, and the transformation of higher education, significant changes have occurred in the nature of academic careers and the working patterns of university teachers. The idealized state of leisurely and serene work has gradually faded. The doors of universities, representing a boundary between the relatively safe and autonomous academic institutional world, and the complex, multi-stakeholder society, literally have to be opened.

For academic staff, this means that, in addition to their regular tasks, they need to develop the willingness and ability to create and foster university-society partnerships, contribute to research agendas, develop real-life learning activities for students and start to collaborate with external stakeholders. Moreover, they have to guide the students through multi-stakeholder research processes and support relevant student competencies [1]. It has become the norm for teachers to face multiple tasks simultaneously [2]. Institutional requirements such as mandated teaching hours, grant completion deadlines, and various meeting schedules collectively constrain faculty time allocation. Limited time resources coupled with multiple burdens of teaching, research, and service obligations create unprecedented levels of stress among academic professionals. This observation aligns with U.S.-based research identifying time pressure and lack of personal time as the most prevalent stressors for faculty members [3]. Moreover, the compounding effects of excessive workloads and disorganized task prioritization not only exhaust practitioners but also diminish productivity, fostering workplace demotivation [4]. According to China’s “Job Burnout Index Survey Report” published by the China Human Resources Development Network, university faculty in China rank third nationally in occupational burnout severity across all industries surveyed [5]. Therefore, in the current environment, effectively managing time conflicts and adjusting the order or priority of time allocation have become crucial for the professional development of teachers [6].

This paper draws on relevant survey data to focus on analyzing the following research questions: What is the work prioritization sequence of university faculty in China under temporal conflicts? How do faculty members’ perceived ideal work sequences differ from their actual practices, and what demographic variations exist in these disparities? What are the underlying mechanisms and causal factors contributing to this mismatch in work prioritization? Based on the identified causes, what targeted interventions can be implemented to promote faculty career development while addressing these prioritization conflicts?

Time is a key concept in both daily life and scholarly pursuits. Although research on time spans various disciplinary traditions, sociology has progressed comparatively slowly in building a knowledge system on time compared to other disciplines [7]. This study therefore offers significant theoretical contributions. Moreover, it provides a fresh perspective. The structuring of time is indicative of the academic profession’s state. Examining the alignment of faculty’s temporal expectations with reality can shed light on the work and life quality of university faculty in China, leading to policy suggestions for their academic growth.

## Literature review

Individuals’ time and task organization in daily life and work is predominantly shaped by two distinct cultural norms and behavioral modes: monochronicity (focusing on one task at a time) and polychronicity (managing multiple tasks simultaneously) [8]. While modern society enables concurrent engagement in multiple activities, practical constraints and organizational demands for time synchronization often constrain individuals to a monochronic work mode, within which the work sequence of university faculty—i.e., their prioritization of various professional tasks—operates as a core research focus.

The existing literature on university faculty’s work sequence presents divergent perspectives, yet these disagreements can be contextualized within a unified analytical framework centered on “task prioritization logic.” Some scholars argue that university faculty’s work lacks a definitive order, characterizing the entire academic system as disorderly [9]. However, a more dominant body of research confirms the existence of inherent task prioritization among university faculty, with discrepancies in conclusions primarily stemming from differences in research contexts and focus. For instance, studies focusing on Chinese universities have identified teaching as the highest-priority task, followed by administrative activities [10], while others, emphasizing academic career development, argue that research takes precedence in faculty work arrangements [11]. These divergent findings collectively reflect that faculty work sequence is not arbitrary but is shaped by context-specific factors.

The prioritization of work sequence and time allocation among university faculty is jointly constrained by individual-level and organizational-social factors, which can be systematically categorized into two theoretical perspectives: psychological and sociological of time.

From a psychological perspective, research emphasizes the role of individual characteristics and psychological factors as endogenous drivers of work sequence. Gender, as a key individual attribute, significantly influences task prioritization: women are found to be primarily engaged in teaching tasks [12], devoting more time to teaching and university service than men, while men allocate more time to research [13]. Beyond gender, psychological factors—including time attitude [14], work motivations [15], and work passion [16]—serve as guiding mechanisms that shape individual task choices and determine work order. Additionally, faculty identity and career status further constrain work arrangements: most professors hold administrative positions (e.g., deans or directors), limiting their time for publications and research grant applications, while associate professors, still in the middle of career advancement (where research performance is prioritized over teaching in evaluation criteria [17]), strive to balance administrative burdens with research efforts.

From the perspective of the sociology of time, faculty work sequence is conceptualized as a non-technical strategy, with its logic shaped by organizational and social environments rather than individual autonomy. German sociologist Hartmut Rosa’s core argument—that “the rhythm, speed, deadlines, and orders of our actions and practices are predetermined by the contractual requirements of collective time patterns in society [18]”—provides a foundational theoretical framework for understanding faculty work arrangements in Chinese higher education. In the organizational context of Chinese universities, the faculty evaluation and assessment mechanism, as an institutionalized form of collective time arrangement, directly embodies Rosa’s “contractual requirements of the social collective time model” and shapes faculty’s work rhythm, task sequence, and time allocation logic. Beyond institutional mechanisms, other organizational and contextual factors—including work nature, organizational development goals, organizational preferences [19], and task performance valence [20]—further influence task prioritization. Notably, even with the same average performance valence, different task sequences and intervals can shape stakeholder perceptions [21], providing faculty with potential flexibility in work order. This flexibility is contingent on work autonomy: when autonomy is low, work sequence is predetermined by managers or processes [22]; in contrast, faculty with extensive research projects, publications, or commercialization experience—who possess greater autonomy—tend to prioritize research over teaching or other tasks [23]. Tenure, as a key institutional factor, also reinforces research prioritization among tenured faculty [23]. However, incomplete individual information and the behaviors of teams, colleagues, supervisors, and customers can all disrupt individual work prioritization [24]. Additionally, task deadlines and urgency serve as universal constraints, as “deadlines are constant, always present in our work and controlling our schedules [25],” and even individuals with low time management disposition will mobilize cognitive resources to complete urgent and important tasks promptly [26].

At a broader social and cultural level, time is a constructed concept transmitted to individuals [7], and modern science and technology has dual impacts on faculty work sequence: while enhancing time utilization efficiency, it also disrupts work rhythms and time order [27]. French philosopher B. Stiegler’s insight in La Technique et le Temps—that “rather than saying that technology is in time, it is more accurate to say that it constructs time [28]”—highlights the transformative role of technology in shaping time structures. Specifically, the intervention of new information technology has severely disrupted the time structure of faculty task sets, potentially reinforcing or significantly altering the sequential or parallel manner in which faculty conduct their work [25], thus adding a new layer of complexity to work sequence dynamics.

## Research design and methodology

### Research design

Regarding the work of university faculty and the scholarship reconsidered, whether it is the classification by Boyer into the scholarship of discovery, application, integration, and teaching [29], or the categorization by Paulsen and Feldman into the scholarship of teaching, service, research and graduate training, academic citizenship [30], scholarship generally involves three aspects: teaching, research, and social service. However, within these seemingly straightforward categories, each contains multifaceted and intricate tasks. Teaching, for instance, extends beyond lesson preparation, class delivery, and post-class feedback; it also includes reflecting on teaching, addressing students’ doubts, providing academic and life guidance, and so on. Research activities, beyond traditional literature review, experimental investigation, academic writing and publication, also involve the management of research projects and teams, as well as coordination among various departments. Compared with teaching and research, social service is more ambiguously defined. Some scholars consider it as management and committee work within the campus [31], while others emphasize its regional feature [32], tending to link it with communities, state and local governments, and business and industry [33].

Therefore, based on the targets and fields of service, it is divided into service for the university (such as various party and government activities, student affairs, etc. at the university, college, or department level), service for external institutions (such as teaching, lectures, consulting and advice at other universities, enterprises, governments, and other institutions), and service for academic disciplines (such as management tasks and peer reviews in various professional organizations or societies). Of course, teaching, research, and social service for university faculty are not isolated from each other; instead, they are interconnected and overlapping.

As noted above, the work of university faculty is complex, and some tasks lack clear definitions even among faculty themselves. Therefore, prior to designing the study’s questionnaire, we conducted semi-structured interviews with more than 40 faculty members from 8 Chinese universities. Based on their responses, we finally identified 10 typical and representative tasks. Specifically, these fall into three categories: in teaching, undergraduate classroom teaching, graduate classroom teaching, undergraduate academic guidance, and graduate academic guidance; in research, academic paper writing and research fund reimbursement; in social service work, international/domestic professional academic meetings, faculty party committee and administrative meetings, university party committee and administrative meetings, and government departmental consultations and meetings. Then, using a questionnaire survey, we asked teachers to rank these tasks from 1 to 10, and compared their ideal work orders with their actual work orders to gain insights into the current situation of academic careers.

As indicated by prior research [34,35], polychoric correlation provides more accurate estimates than Pearson correlation for ordinal data, especially with skewed distributions. Therefore, this study used Mplus software, and the analysis revealed that the data can be divided into three major factors: teaching, research, and social service. The composite reliability (CR) of each factor was greater than 0.6, the root mean square residual (RMR) was less than 0.08, and the goodness-of-fit index (GFI), adjusted goodness-of-fit index (AGFI), and Tucker-Lewis index (TLI) were all greater than 0.9, indicating that the data has good reliability and validity(see Table 1 for details).

**Table 1:** Reliability and validity analysis of the data.

Factor	CR (Composite Reliability)	RMR	GFI	AGFI	TLI
Teaching	0.939	0.031	0.999	0.991	0.997
Research	0.760	0.057	0.999	0.986	0.970
Social Service	0.744	0.070	0.999	0.984	0.991

In terms of relevant influencing factors, based on the literature review and relevant interview data, this study mainly explores these influencing factors from two dimensions: individual factors and university organizational factors. Individual factors include individual innate characteristics (such as gender and age), identity and status (such as professional title, administrative position, academic part-time jobs, etc.), and psychological factors (such as career aspiration, etc.). University organizational factors include institutional academic reputation, discipline reputation, and the university management system. The specific research framework is shown in Fig 1.

**Fig 1:**
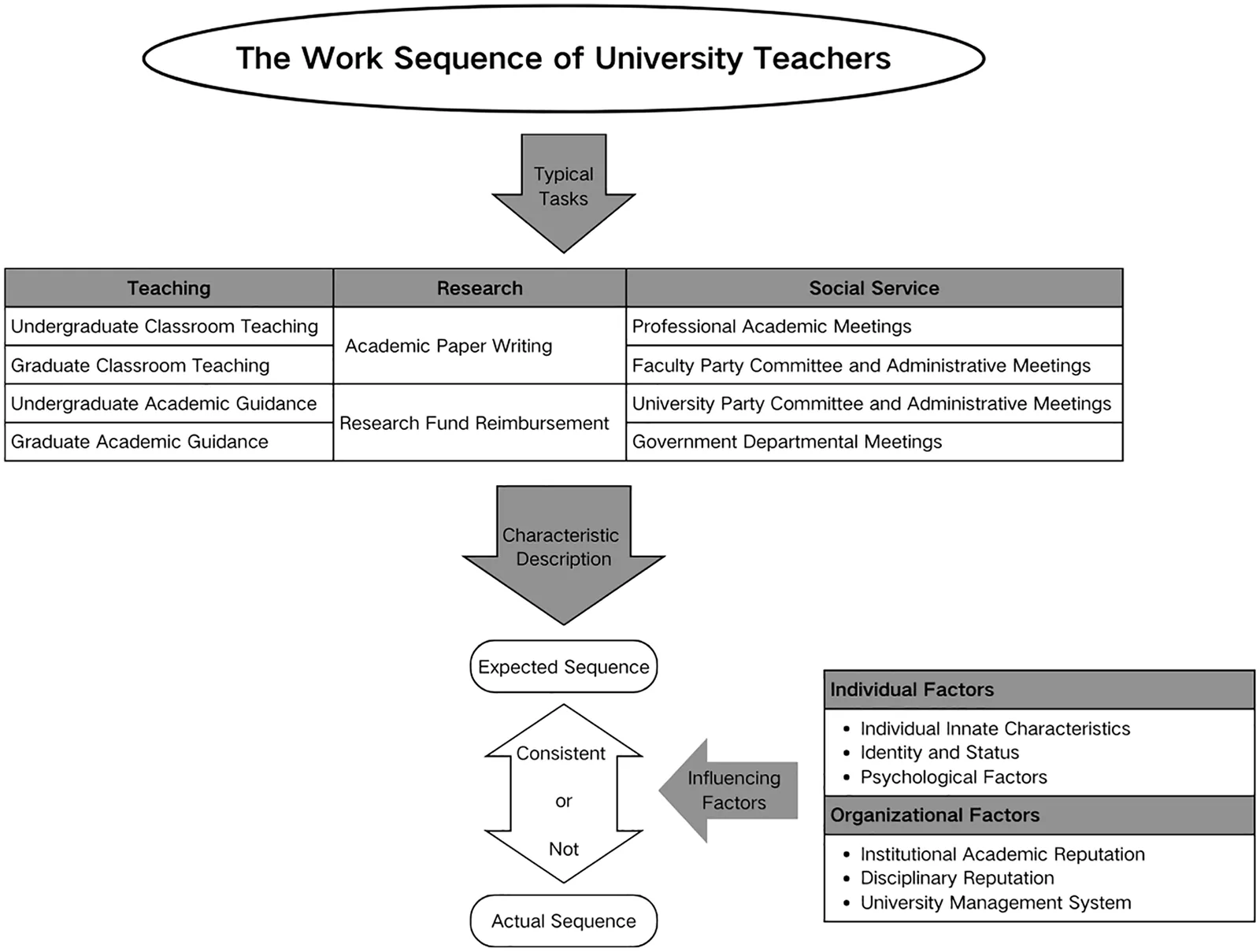
Research framework.

### Data description

Given the distinct operational characteristics of vocational institutions, this study focuses exclusively on faculty from general undergraduate universities in China. To ensure methodological rigor, a stratified sampling approach was adopted by region. According to 2021 statistics from the Ministry of Education of China, there were 1,238 undergraduate-level higher education institutions (excluding vocational colleges at the undergraduate level), with approximately 1.2697 million full-time faculty. Among them, teachers in the eastern region (including northeastern China) accounted for about 61.7%, the central region for 18.5%, and the western region for 19.8%. To further enhance representativeness, disciplinary distribution was also considered during sampling (note: military and medical disciplines were excluded due to their specificity).

Combined with the realistic characteristics of China’s higher education, such as unbalanced regional development, hierarchical layout of institutions, and diversified discipline types, this study adopted a three-dimensional composite stratified sampling design (see Table 2 for details). First, according to the differences in the distribution of China’s higher education resources, it was divided into three major regions: Eastern, Central, and Western China; second, within each region, it was divided into three categories: national first-class universities, first-class universities in the respective provinces, and other general institutions; finally, within institutions of the same region and same level, the discipline categories were subdivided into humanities, social sciences, natural sciences, and engineering technology, and the sample size was allocated according to the overall structural proportion. Although this study ensured the standardization of statistical stratified sampling to the greatest extent, in reality, not all teachers filled in the questionnaire, so the effective sample size may deviate from the actual situation. In addition, due to the complex setting of colleges and departments within each school and the impact of the epidemic at that time, some links failed to achieve completely accurate standardized sampling, resulting in minor deviation defects. However, the overall sample coverage is sufficient and the core stratified structure is complete; therefore, the survey data of this study still has good overall representativeness, which can support subsequent overall data analysis, law research, and the derivation and interpretation of relevant conclusions.

**Table 2:** Sampling distribution of this study.

Region	City	Number of Institutions	Distribution of Institution Levels	Discipline Distribution
Eastern China	Beijing	5	• National First-Class Institutions (2) • Provincial/Municipal First-Class Institutions (2) • Other General Institutions (1)	Covering all disciplines, including 4 National First-Class Disciplines, 5 Provincial/Municipal First-Class Disciplines, and 9 Other General Disciplines.
	Shanghai	5	• National First-Class Institutions (2) • Provincial/Municipal First-Class Disciplines (1) • Other General Institutions (2)	Covering all disciplines, including 3 National First-Class Disciplines, 6 Provincial/Municipal First-Class Disciplines, and 7 Other General Disciplines.
	Jiangsu	4	• National First-Class Institutions (2) • Provincial/Municipal First-Class Institutions (1) • Other General Institutions (1)	Covering all disciplines, including 4 National First-Class Disciplines, 6 Provincial/Municipal First-Class Disciplines, and 6 Other General Disciplines.
	Liaoning	3	• National First-Class Institutions (1) • Provincial/Municipal First-Class Institutions (1) • Other General Institutions (1)	Covering Philosophy, Literature, Economics, Education, Agronomy, Natural Sciences, and Engineering, including 1 National First-Class Discipline, 2 Provincial/Municipal First-Class Disciplines, and 4 Other General Disciplines.
	Fujian	3	• National First-Class Institutions (1) • Provincial/Municipal First-Class Institutions (1) • Other General Institutions (1)	Covering Economics, Law, Literature, History, Natural Sciences, Engineering, Agronomy, Management, and Art, including 1 National First-Class Discipline, 3 Provincial/Municipal First-Class Disciplines, and 6 Other General Disciplines.
Central China	Jiangxi	3	• Provincial/Municipal First-Class Institutions (2) • Other General Institutions (1)	Covering all disciplines, including 4 Provincial/Municipal First-Class Disciplines and 5 Other General Disciplines.
	Hubei	4	• National First-Class Institutions (1) • Provincial/Municipal First-Class Institutions (1) • Other General Institutions (2)	Covering all disciplines, including 3 National First-Class Disciplines, 4 Provincial/Municipal First-Class Disciplines, and 6 Other General Disciplines.
	Hunan	4	• National First-Class Institutions (1) • Provincial/Municipal First-Class Institutions (1) • Other General Institutions (2)	Covering all disciplines, including 2 National First-Class Disciplines, 5 Provincial/Municipal First-Class Disciplines, and 7 Other General Disciplines.
Western China	Sichuan	4	• National First-Class Institutions (1) • Provincial/Municipal First-Class Institutions (2) • Other General Institutions (1)	Covering all disciplines, including 3 National First-Class Disciplines, 5 Provincial/Municipal First-Class Disciplines, and 6 Other General Disciplines.
	Chongqing	2	• National First-Class Institutions (1) • Other General Institutions (1)	Covering all disciplines including Philosophy, Law, Literature, History, Natural Sciences, Engineering, Agronomy, Management, and Art, including 2 National First-Class Disciplines and 7 Other General Disciplines.
	Shaanxi	5	• National First-Class Institutions (1) • Provincial First-Class Institutions (2) • Other General Institutions (2)	Covering all disciplines, including 2 National First-Class Disciplines, 3 Provincial/Municipal First-Class Disciplines, and 6 Other General Disciplines.
	Yunnan	2	• Provincial/Municipal First-Class Institutions (1) • Other General Institutions (1)	Covering Philosophy, History, Economics, Law, Education, Natural Sciences, and Engineering, including 2 Provincial/Municipal First-Class Disciplines and 5 Other General Disciplines.

By January 2022, we collected a total of 1,232 valid questionnaires from faculty at 44 institutions across 12 Chinese provinces and municipalities. Analysis shows that respondents from eastern, central, and western regions constituted 61.1%, 18.6%, and 19.8% of the sample, respectively, which aligns closely with the national distribution of university faculty and supports the scientific validity of the data.

Detailed sample characteristics are presented in Table 3.

**Table 3:** Effective percentage of samples.

Basic Information	Sample Category	Percentage
Gender	Male	57.5
	Female	42.5
Age	35 and under	26.1
	36–45	38.1
	46–55	25.3
	56 and above	10.5
Administrative Position	Yes	23.9
	No	76.1
Part-time Academic Position	Yes	24.8
	No	75.2
Graduate Academic Advisor	Yes	47.3
	No	52.7
Region	Eastern China	61.1
	Central China	18.6
	Western China	20.3
Marital Status	Married	83.6
	Unmarried	16.4
Academic Rank	Full Professor	21.9
	Associate Professor	39.0
	Lecturer or Below	39.1
Discipline	Humanities	22.4
	Social Sciences	31.7
	Natural Sciences	17.1
	Engineering and Technology	28.9
Discipline Tier	National “Double First-Class”	18.3
	Provincial/Municipal “Double First-Class”	21.5
	Other General Disciplines	60.2
Institutional Academic Reputation	National “Double First-Class” University	28.9
	Provincial/Direct-Controlled Municipality “Double First-Class” University	24.8
	Other General Undergraduate Institutions	46.3

Note: Humanities include philosophy, literature, history, and arts; Social sciences include economics, law, education, and management; Natural sciences refer to science disciplines; others belong to engineering and technology.

## Research findings

### General characteristics of teachers’ work sequence

Descriptive analysis (Table 4) shows that classroom teaching, especially undergraduate teaching, holds a relatively high priority in both faculty’s ideal expectations and actual work scenarios, with its mean and mode ranking among the top three. The expected and actual order of student academic guidance, research funding reimbursement, and academic conferences are relatively consistent, about 4–6. However, there is a stark contrast between the ideal and actual work order of academic paper writing and various party committee and governmental meetings: in the expected order, academic paper writing ranks relatively high, both in terms of its mean and mode. Yet, in actual work situations, its average order falls below 6, and even many teachers rank it last (with a mode of 10). Conversely, the order for various party committee and governmental meetings is the opposite.

**Table 4:** Mean and mode of the order of various tasks for teachers.

	Mean	Mode		
	Expected Order	Actual Order	Expected Order	Actual Order
Undergraduate Classroom Teaching	2.10	1.71	1	1
Graduate Classroom Teaching	3.48	3.13	2	2
Undergraduate Academic Guidance	4.78	4.82	5	3
Graduate Academic Guidance	4.62	5.20	4	4
Academic Paper Writing	3.17	5.83	1	5
Research Fund Reimbursement	6.45	6.63	7	6
Professional Academic Meetings	5.69	6.43	6	7
Faculty Party Committee and Administrative Meetings	7.80	5.78	8	5
University Party Committee and Administrative Meetings	8.12	5.80	9	9
Government Departmental Meetings	8.33	6.52	10	10

When ranking the top three as high priority and the bottom three as low priority, frequency analysis (Fig 2) yields result consistent with the above findings. Most faculty members consider classroom teaching a high priority, both ideally and in practice. Specifically, 83.2% ideally and 89.6% actually rank undergraduate teaching among their top three tasks, with 55.6% and 73.6% placing it first. This reflects the increasing institutional emphasis on teaching—particularly undergraduate instruction—in recent higher education evaluation reforms, with many universities restricting revisions to undergraduate courses. Regarding student advising, a notable proportion of faculty ideally prioritize undergraduate (45.9%) and graduate (28.9%) advising, and in practice place them within the top three (33.6% and 21.8%, respectively). However, fewer than 5% rank either as their top task (0.5% for undergraduate, 2.2% for graduate). Moreover, nearly 20% assign advising—especially graduate advising (17.8%)—the lowest priority, indicating room for strengthening its institutional value.

**Fig 2:**
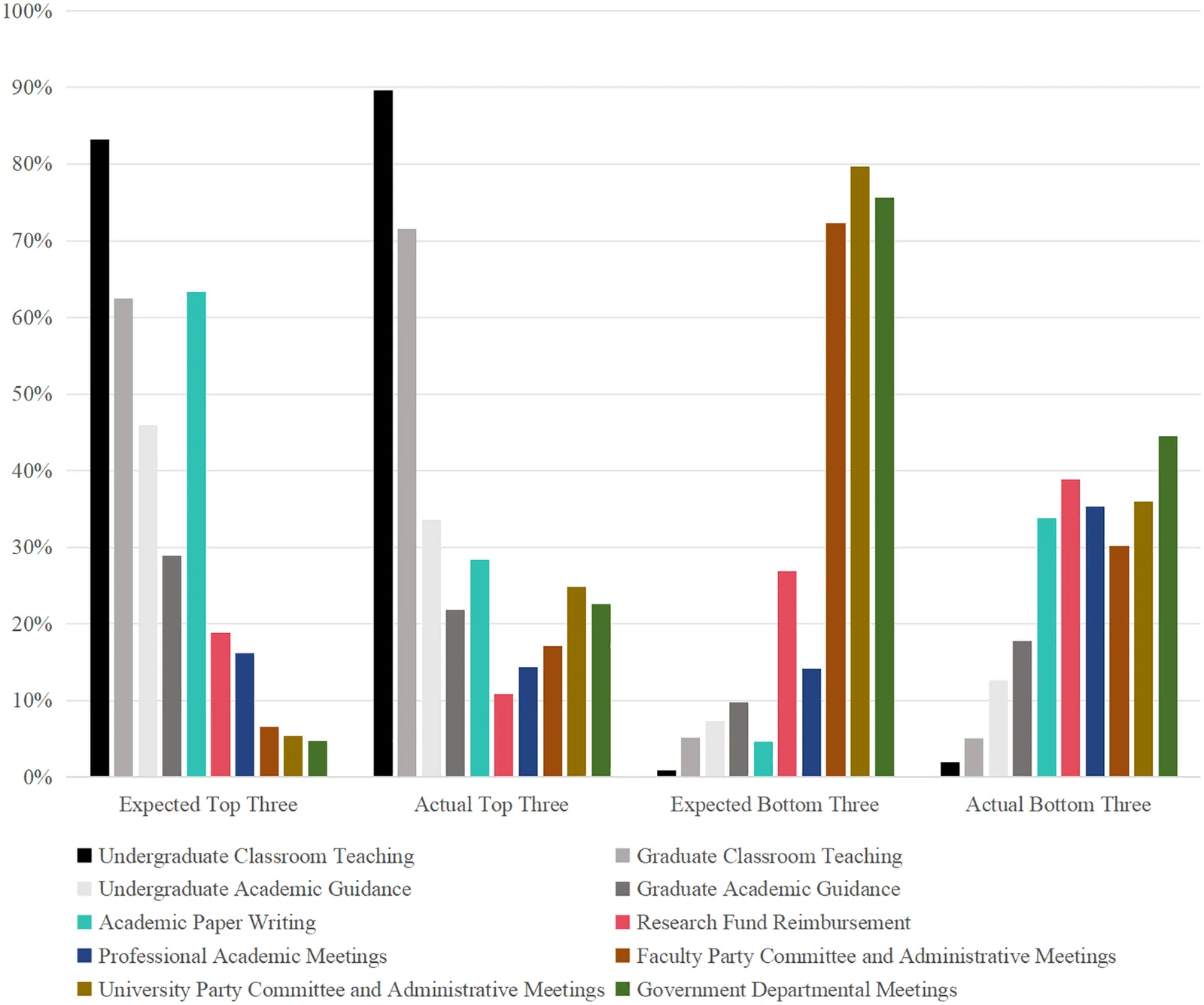
Percentage of faculty ranking tasks in the top/bottom three.

Similarly, while over 60% of faculty ideally prioritize academic writing, fewer than 30% actually rank it among their top three when tasks conflict. In contrast, substantial proportions prioritize political-administrative meetings at the departmental (17.1%), university (24.8%), and government (22.6%) levels.

### Analysis of discrepancies in faculty prioritization of academic versus political-administrative work within the “Double First-Class” initiative framework

Given the significant discrepancy between the ideal and actual rankings of academic writing and political-administrative meetings, this section analyzes the magnitude of these discrepancies and their influencing factors. Aligned with the survey scale, The difference between the actual order and the expected order (an integer ranging from −9–9) was classified into five levels: specifically, a difference within the range of [−9, −6] was recorded as 1 (indicating that the actual order is much lower than the expected order); a difference within the range of (−6, −1) was recorded as 2; a difference within the range of [−1, 1] was recorded as 3 (indicating that the two orders are roughly consistent); a difference within the range of (1, 6) was recorded as 4; and a difference within the range of [6,9] was recorded as 5 (indicating that the actual order is much higher than the expected order). The three types of political-administrative meetings are grouped, and their average ranking is used. Table 3 presents the percentage distribution of discrepancies between actual and ideal rankings of academic writing and political-administrative tasks. Regarding academic writing, nearly half (48.8%) of the faculty rank it lower in practice than ideally. In contrast, over 40% prioritize political-administrative tasks higher in actual practice than they would prefer (Table 5).

**Table 5:** Effective percentage distribution of discrepancy between actual and expected rankings for academic writing and political-administrative meetings.

	The actual order is far behind the expected order.	The actual order is slightly behind the expected order.	The actual order is slightly ahead of the expected order.	The actual order is far ahead of the expected order.
Academic Paper Writing	0.1	3.4	23.2	25.6
Various Political-Administrative Meetings	5.1	35.6	5.2	0

Further analysis (as shown in Table 4) reveals that regarding institutional academic reputation, faculty at universities selected for provincial/municipal “first-class university” initiatives express a stronger preference for prioritizing academic work (mean = 2.8), showing a significant difference (P < 0.05) compared with other regular institutions. However, the actual ranking of academic work among these faculty is notably lower (mean = 6).

From a disciplinary perspective, faculty in disciplines selected for provincial/municipal “first-class discipline” programs also report a higher preferred priority for academic work (mean = 2.84). Yet in practice, higher disciplinary prestige correlates with a lower actual ranking of academic tasks. This discrepancy is closely linked to the competitive framework of the “Double First-Class” initiative, which operates on a five-year review cycle with upward and downward mobility. While national “world-class university” status remains relatively stable—largely retained by former “985” institutions—provincial-level “first-class universities” still have opportunities to advance to the national tier. Given that academic excellence is a key criterion for national recognition, many provincial-level institutions intensify research expectations, thereby transferring considerable pressure onto faculty, who consequently express a stronger desire to prioritize academic work.

A similar dynamic applies to “world-class discipline” selection, which involves a broader range of institutions and more available slots. Even non-elite universities can qualify for national “world-class discipline” status, prompting many to reinforce disciplinary development and emphasize scholarship. As a result, faculty in these institutions and disciplines likewise feel compelled to prioritize academic activities (Table 6).

**Table 6:** Differences in faculty prioritization of academic work based on institutional and disciplinary reputation.

			Selected for National “Double First-Class” Initiative	Selected for Provincial/Municipal “Double First-Class” Initiative	Other General Institutions
Institutional Academic Reputation	Expected Order	Mean	3.32	2.80	3.27
		Std.Dev	2.221	2.142	2.046
		F	6.188		
		P	0.002		
	Actual Order	Mean	5.85	6.00	5.72
		Std.Dev	2.783	3.014	3.024
		F	0.9		
		P	0.407		
Disciplinary Reputation	Expected Order	Mean	3.04	2.84	3.33
		Std.Dev	2.347	2.026	2.084
		F	5.742		
		P	0.003		
	Actual Order	Mean	6.2	6.02	5.65
		Std.Dev	2.976	3.003	2.918
		F	3.671		
		P	0.026		

Table 7 presents the ranking of political-administrative tasks among faculty.** ANOVA results indicate that faculty at institutions and disciplines selected for national or provincial/municipal “Double First-Class” initiatives rank these tasks significantly lower in their preferred order compared to their counterparts at regular institutions (p < 0.005). However, no significant difference is observed in the actual ranking across the three groups (p > 0.05). In other words, regardless of institutional or disciplinary prestige, faculty prioritize political-administrative meetings similarly in practice, with mean rankings averaging around the sixth position.

**Table 7:** Differences in faculty ranking of political-administrative meetings based on institutional and disciplinary reputation.

			Selected for National “Double First-Class” Initiative	Selected for Provincial/Municipal “Double First-Class” Initiative	Other General Institutions
Institutional Academic Reputation	Expected Order	Mean	8.20	8.24	7.92
		Std.Dev	1.286	1.300	1.639
		F	6.069		
		P	0.002		
	Actual Order	Mean	6.49	6.43	6.28
		Std.Dev	2.217	2.250	2.307
		F	0.786		
		P	0.456		
Disciplinary Reputation	Expected Order	Mean	8.26	8.31	7.95
		Std.Dev	1.233	1.177	1.602
		F	7.771		
		P	0.001		
	Actual Order	Mean	6.03	6.54	6.43
		Std.Dev	2.201	2.287	2.270
		F	2.684		
		P	0.069		

(Note: The value for political-administrative meetings = (departmental meetings + university-level meetings + government-level meetings)/ 3).

### Factors influencing the imbalance between academic and party-government work priorities

What factors underlie this phenomenon? Following the analytical framework outlined earlier. Multiple linear regression (MLR) was employed to examine this issue. The analysis indicated that both models (Model 1: the gap between actual and expected priority of academic paper writing; Model 2: the gap between actual and expected priority of various Party-government meetings/activities) were statistically significant (P < 0.005).

As shown in Table 8, among individual factors, personal ascribed characteristics (e.g., gender, age) have no significant influence on the discrepancy between actual and ideal priorities for either academic writing or Party-government work (p > 0.05). While professional title and administrative position show no significant effect on the priority gap concerning academic writing (p > 0.05), they significantly affect the discrepancy in Party-government work priorities (p < 0.05). Specifically, higher professional titles are associated with a greater actual than expected priority given to Party-government work. Furthermore, teachers holding administrative roles prioritize Party-government tasks over academic work when conflicts arise, compared to those without such responsibilities. Regarding psychological factors, academic idealism significantly influences the ideal-actual discrepancy in both academic and Party-government work priorities (p < 0.05). Teachers with stronger academic aspirations tend to prioritize academic tasks and defer Party-government affairs. Conversely, a significant negative correlation exists with achievement motivation in administrative management and social service (p < 0.05); teachers more motivated to excel in administration assign higher actual than expected priority to such work.

**Table 8:** Results of multivariate linear regression on the gap between actual and expected priorities for teachers’ academic writing and party-government meeting activities.

		Independent Variable	Parameter Estimates for Model 1		Parameter Estimates for Model 2	
			Beta Coefficient	P	Beta Coefficient	P
Individual Factors	Innate Characteristics	Gender (male = 1, female = 0)	−0.016	0.572	−0.034	0.310
		Age	0.046	0.212	0.030	0.485
	Identity and Status	Job Title (decreasing from 1 to 4)	0.123	0.594	0.123^*^	0.011
		Administrative Position (1 = yes, 0 = no)	0.007	0.815	−0.125^*^	0.001
		Academic Part-time Job (1 = yes, 0 = no)	0.015	0.648	0.013	0.725
		Academic Advisor (1 = yes, 0 = no)	0.011	0.776	0.043	0.308
		Part-time Management Role (1 = yes, 0 = no)	−0.012	0.685	−0.002	0.947
	Work Aspiration	Academic Aspiration (decreasing from 1 to 5)	−0.138^***^	0.001	−0.125^***^	0.004
		Party-Government Work Aspiration (increasing from 1 to 5)	−0.040	0.233	0.116^***^	0.003
Organizational Factors	Institutional and Disciplinary Reputation	Institutional Academic Reputation (decreasing from 1 to 3)	0.150^**^	0.002	−0.145^*^	0.010
		Disciplinary Reputation (decreasing from 1 to 3)	−0.092^**^	0.006	0.087^*^	0.027
	Management System	Level of Institutional Emphasis on Academics (decreasing from 1 to 5)	−0.054	0.115	−0.096^*^	0.032
		Degree of Administrative Intervention (increasing from 1 to 5)	0.059	0.064	0.062	0.066

Note: ^*^ indicates P < 0.05; ^**^ indicates P < 0.01; ^***^ indicates P < 0.005.

At the institutional level, both university academic prestige and disciplinary reputation are significant factors affecting the ideal-actual priority gap for academic writing and Party-government work (p < 0.05). Higher institutional academic prestige correlates with a lower actual than expected priority for academic work and a more deferred priority for Party-government tasks, whereas the influence of disciplinary reputation operates in the opposite direction. Similarly, within management systems, greater institutional emphasis on academic work corresponds to a lower actual priority for Party-government tasks, resulting in a wider gap between actual and expected sequences (regression coefficient = −0.096). Additionally, although not statistically significant (P = 0.066), the degree of administrative intervention within the university shows a tendency to weaken the priority given to academic work.

## Conclusion and discussion

### Research conclusions

First, undergraduate teaching holds an absolute priority, a finding consistent with numerous prior studies [36–43]. Academics themselves are the main drivers towards a strong research role, whereas the regulatory context continues to opt for a dominant teaching role [44]. With the implementation of the “Double First-Class” initiative in recent years, China has consistently emphasized strengthening undergraduate education and issued a series of national undergraduate teaching policies. For instance, in 2019, the Ministry of Education issued the “Opinions on Deepening the Reform of Undergraduate Education and Teaching to Comprehensively Improve the Quality of Talent Cultivation”, followed by the “Overall Plan for Deepening the Reform of Educational Evaluation in the New Era” in 2024. Both documents explicitly emphasize the central role of undergraduate teaching. In the vast majority of higher education institutions, teaching time is pre-determined as institutional time, which is formalized in the form of course schedules and serves as an organizational rule that all university members must adhere to. Therefore, whether it is what teachers expect or what actually happens in real work scenarios, undergraduate teaching has absolute priority.

But studies have shown that while a high proportion of academic staff participates in undergraduate teaching, levels of student satisfaction are low. The main causes of this situation are: a shortage of well-qualified academic staff; the pressure on academic staff to focus their time on research output; practices that emphasize research-orientation in the actual evaluation of academic staff performance; and poor support for teaching in terms of both support staff and teaching facilitie [45]. Moreover, in the USA, a close linkage of teaching and research often was viewed as appropriate for the sector of graduate education, but not for undergraduate education [44]. The reality, however, is that every newly appointed faculty member begins their career with undergraduate teaching obligations, and must demonstrate research productivity through publications and grants to secure career advancement—and subsequently, eligibility for graduate education and instruction. Currently, driven by both institutional requirements for faculty competency and individual career development aspirations, there exists no academic role exclusively dedicated to undergraduate teaching alone. For university faculty, teaching and research are the two indispensable components of academic careers. Under the weight of multiple responsibilities and the reality of “up-or-out” tenure policies, while undergraduate teaching retains its nominal primacy, such institutional prioritization merely secures procedural compliance, with pedagogical quality often being compromised in practice.

Secondly, management activities enjoy a higher priority in scheduling compared with scientific research. Time is a symbolic product collectively shaped and defined by humans at a deep level, serving the needs of human coordination and meaning-making [46]. The question of who determines the rhythm, duration, speed, order, and synchronization of events and actions constitutes a core area of conflict of interests and power struggles [18]. When the demands of different time subjects’ conflict, the underlying power relations often play a decisive role. Generally speaking, university power mainly comprises academic power and administrative power. Academic power is derived from the academic logic of “advanced and profound knowledge,” emphasizing freedom and autonomy. Administrative power, on the other hand, is a power structure based on rational behavior, derived from the legal-rational logic of bureaucratic organizations, emphasizing efficiency and hierarchy. Due to the differences in their power sources and value concepts, contradictions and conflicts often arise during the operational management of universities, one manifestation of which is the generalization of administrative power.

Under the influence of the Double First-Class initiative, many universities have begun to prioritize academic work, and the level of administrative intervention in faculty research has moderated—consistent with this study’s finding that administrative power does not significantly affect the prioritization of academic tasks. However, influenced by historical factors and cultural environments of university development, Chinese universities are more accustomed to managing themselves according to the organizational principles and logic of government administrative institutions. Coupled with the fact that academic resources and decision-making power are largely in the hands of administrative departments, administrative power often prevails in the internal management of universities. Even though faculty expect various Party and government meetings to be placed relatively low in priority, these activities nevertheless retain a comparatively higher position in actual practice. As noted by scholars, administrative forces have given relative priority to management activities in Chinese universities, and university administrators do not regard research engagement as a legitimate excuse for absence from such activities [10].

While this scheduling facilitates the efficient operation of administrative affairs in universities—ensuring smooth teaching order, resource allocation, and other managerial processes—it also risks undermining the core institutional mission of talent cultivation and knowledge innovation. Because when administrative affairs take precedence, they tend to exacerbate bureaucratic tendencies within academic institutions, distorting faculty priorities and severely undermining both the quality of education and scientific research output.

Third, faculty place significantly higher expected priority on research time than its actual position in their workflow. Influenced by the evaluation mechanism for university teachers and the academic promotion system, research has become increasingly important in teachers’ work. Moreover, scientific research demands slow and sustained accumulation through long-term, uninterrupted cycles (e.g., 3–5 years of stable support) to foster systemic innovation, rather than merely chasing short-term metrics. The world’s longest-running experiments reveal that scientific progress often resembles a marathon [47], not a sprint with instant outcomes. Especially in the context of Double First-Class construction, many universities striving for national listing further emphasize academic research, leading faculty in institutions yet to achieve this status to desire greater precedence for scholarly work. Nevertheless, since research time is largely self-managed by faculty, the lack of external institutional safeguards results in fragmented and continually deferred research hours. Consequently, “busy with administrative tasks during weekdays/working hours, and conducting research at night/during holidays” has become the norm for many teachers, and “working overtime” becomes a common phenomenon in the work of university teachers. This study found that teachers invest approximately 27 hours per week (including weekends) in research (M = 27.32, S.td = 18.403), accounting for more than half of their total working time (M = 52.57, S.td = 20.315). Among them, over 85% of teachers conduct research activities after 8 PM (88.6%) or during weekends and holidays (86.6%).

In recent years, despite the introduction of various policies in China to promote diversified evaluation mechanisms for university faculty, the prevailing emphasis on research—driven by university rankings, the “Double First-Class” initiative, disciplinary evaluations, and talent programs—has made research a core task and a key factor for career advancement. When research time is consistently deprioritized, it directly leads to insufficient time and energy for in-depth research projects and scholarly inquiry. This severely constrains research output and the enhancement of academic influence, undermines institutional competitiveness and innovation capacity in academia, and ultimately impedes individual faculty members’ scholarly growth and professional development.

### Relevant implications

Education is the foundation of a country’s prosperity, and teachers are the cornerstone of educational development. To create a world-class higher education system, priority should be given to developing a healthy and vibrant faculty body. Therefore, in order to deal with a series of consequences caused by the unsatisfactory work sequence of university and college teachers under the time conflict, multi-targeted measures should be implemented to promote the healthy and professional development of teachers.

Macro-level: Governments and relevant education authorities should take a leadership role in continuously developing and improving supportive policies and institutional systems to safeguard faculty development. On the one hand, legal frameworks should be strengthened to protect faculty’s professional status and legitimate rights. Guided by the Constitution, the teacher law and education law should establish institutional safeguards for protecting faculty’s legitimate rights and interests. Meanwhile, legal provisions should affirm faculty’s central role in higher education institutions and treat faculty development as an institutional priority, thereby effectively safeguarding faculty’s rights to professional development. On the other hand, faculty evaluation policies should be further refined to focus on the substantive professional development of faculty. Although China has consistently prioritized the reform of faculty evaluation systems—for instance, the Ministry of Education issued the “Guidelines on Deepening the Reform of Faculty Evaluation Systems in Higher Education Institutions” in August 2016, and the Central Committee of the Communist Party and the State Council released the “Overall Plan for Deepening Education Evaluation Reform in the New Era” in October 2020—the research-biased evaluation paradigm persists in the current environment that favors various talent programs and research-oriented resource competition. Some institutions have merely superimposed teaching evaluations onto existing research-oriented criteria, which inadvertently increases faculty’s teaching burdens. Therefore, the government should intensify efforts to reform faculty evaluation systems, placing greater emphasis on substantive evaluation of faculty development.

Meso-level: higher education institutions and relevant departments should promptly establish sound management mechanisms to ensure efficient administrative operations and protect faculty’s academic time through institutional arrangements. As faculty represent the main body of university development, institutional organization and all administrative activities should be academic-centered and service-oriented. Institutions should effectively guarantee and implement faculty’s academic authority, clearly delineate academic versus non-academic responsibilities, and restore time that rightfully belongs to academic work. Moreover, it is not only the concern for an appropriate curriculum and the scope of knowledge transmitted that are important factors for the development of university staff. Also crucial is the need to provide teachers with many-sided opportunities for their own development. University teachers are also largely active academics, conducting research and having contact with the business community. The multi-dimensional nature of this work means that the direction and scope of the areas needed to be supplemented is an individual matter [48]. Therefore, special attention should be paid to the personalized career development of early-career faculty, providing them with sufficient and flexible working time to explore their areas of expertise and inspiring their conscious and proactive professional growth.

Micro-level: faculty members should enhance self-management, pay attention to their work conditions, and improve both professional and life quality. Time management is defined as “a form of decision making used by individuals to structure, protect, and adapt their time to changing conditions” [49]. Therefore, one of the core dimensions of time management measurement is “time protection”. And one of the items reflecting time protection is “Do you often find yourself doing things which interfere with your schoolwork simply because you hate to say ‘No’ to people?” [50] Therefore, specifically, the practical forms of time protection include actively saying “No” to time-consuming and unnecessary requests from colleagues, friends, and others, or reducing the occupation of one’s own time by non-core affairs through methods such as setting exclusive working hours and turning off interference sources. Its core implication is that individuals should set boundaries around their own time to resist various irrelevant interferences and further ensure the progress of core tasks. Accordingly, in order to further improve their own time awareness, strengthen time management, and use time to the maximum and most effective extent, teachers should sometimes learn to refuse non-essential tasks to ensure sufficient autonomous time.

Finally, it should be noted that since the data was collected during the epidemic period, affected by COVID-19, the lives of Chinese university teachers were subject to certain disturbances, which may impose certain limitations on the generalizability of the results and their promotion and application in normal scenarios. In addition, the countermeasures and suggestions proposed in this study are mainly based on theoretical analysis and deduction, and there may be limitations in adaptability and operability in practical implementation. In the follow-up, this study will continue to deepen and track, so as to further improve data supplementation and achievement optimization.

### Ethics statement

This study was conducted in strict accordance with the ethical principles of the Helsinki Declaration. Before commencing the survey, all participants were clearly informed of the research purpose, content, and procedures to ensure their full understanding. Each participant provided written informed consent voluntarily prior to participation.

The survey does not involve any personal privacy content. The questionnaire was administered anonymously, and all collected data will be used solely for aggregated academic analysis. Participants’ personal information will be kept strictly confidential; no personally identifiable information will be recorded, stored, or disclosed. Data will be processed in encrypted and aggregated form during storage and analysis to ensure security.

The rights, safety, and well-being of participants are our foremost priority throughout the research process. We are committed to adhering to the above ethical principles at all stages of the study.

## Supporting information

S1 File Informed consent form. (DOCX)

S2 File Original database. (XLSX)
