# Supplementary material for: Requisitioned academic time: A contradictory deconstruction of Chinese university faculty’s work priorities under the “Double First-Class” initiative
Source: PLoS One. 2026 Sep 29;21(9):e0356772. doi: 10.1371/journal.pone.0356772 (PMC13623128; doi:10.1371/journal.pone.0356772)
Supplement: Supplementary file 1 [file pone.0356772.s001.docx]

| **Informed Consent Form** | | | | | |
| --- | --- | --- | --- | --- | --- |
| **Research Title** | Requisitioned academic time: a contradictory deconstruction of Chinese university faculty’s work priorities under the “Double First-Class” initiative | | | | |
| **Principal Investigator** | FU Mengyun | **Tel** | 13606043917 | **Email** | jjfumengyun@126.com |
| **University** | Huaqiao University | **College** | Chinese Language and Cultural College | **Academic title** | Lecturer |
| Dear Participant,  We sincerely invite you to participate in the research project entitled Requisitioned academic time: a contradictory deconstruction of Chinese university faculty’s work priorities under the “Double First-Class” initiative. Before deciding whether to participate, please carefully read the following information. This Informed Consent Form aims to explain the purpose, process, potential risks, and protective measures of the research, ensuring that you voluntarily participate based on a full understanding.  This research will strictly adhere to relevant ethical norms throughout the entire process, fully protecting your right to information and safety during participation. | | | | | |
| **1. Research Background and Objectives**  **Research Background**  With the development of modern information technology, popularization of the internet, and advancement of the “Double First-Class” Initiative in China, the nature of academic professions and the work methods of university faculty have undergone profound transformations. The leisure-oriented state of traditional academic work has gradually disappeared. In addition to undertaking conventional teaching and research tasks, faculty members are also required to take on new responsibilities such as social services, university-enterprise cooperation, student guidance, and participation in research involving multiple stakeholders. The parallel execution of multiple tasks has become the norm. Meanwhile, factors such as class hour requirements, research project completion deadlines, and meeting arrangements have further compressed faculty members’ time and space, making time conflicts a widespread issue. Therefore, how to balance task priorities and resolve time conflicts has become a key topic affecting the professional development of university faculty.  **Research Objectives**  Based on the current situation of time conflicts faced by Chinese university faculty, this research aims to explore four core issues using survey data: 1) the actual work prioritization of university faculty under time conflicts; 2) the differences between faculty members’ personal expected work order and their actual prioritization; 3) the influencing mechanisms and underlying reasons for the inconsistency between expected and actual prioritization; 4) targeted measures to promote faculty professional development based on the analysis of these reasons. Ultimately, this research intends to provide practical references for the healthy and sound development of academic professions in Chinese universities, as well as a theoretical basis and practical insights for the formulation and optimization of relevant policies. | | | | | |
| **2. Research Methods and Procedures**  **Research Participants**  University faculty members from multiple universities in China, with an expected sample size of approximately 2,000 participants, covering universities in no fewer than 12 provinces/municipalities directly under the Central Government.  **Research Methods**  Primarily anonymous questionnaire surveys, with some participants invited to participate in supplementary interviews (all on a voluntary basis).  **Research Procedures**  - You will complete an anonymous questionnaire including basic background information and questions related to work prioritization under time conflicts;  - The estimated time required to complete the questionnaire is 6-10 minutes;  - The research period is from October 2021 to January 2026. | | | | | |
| **3. Potential Benefits and Risks of the Research**  **Potential Benefits**  It helps you understand the actual status of academic professions in Chinese universities and gain a deeper insight into the nature of academic careers. Additionally, the research results will contribute to optimizing your time management and enhancing your work efficiency.  **Potential Discomfort or Risks**  Participating in this questionnaire survey will not cause you any harm. Due to the relatively large number of questions in the questionnaire, it may take up some of your leisure time, but this will not affect your normal work and life. | | | | | |
| **4. Participants’ Rights and Information Protection**  -To safeguard your rights, interests, and personal privacy, this research makes the following commitments:  **Right to Voluntary Participation and Withdrawal**  -Your participation is entirely voluntary. You may terminate the questionnaire at any time during the research without providing a reason;  -You have the right to agree to or decline participation in any part of the research.  **Confidentiality of Personal Information**  If you participate in this research, your personal information (such as address, phone number, date of birth, etc.) and opinions will be kept confidential. Your responses to the questionnaire will be anonymized and aggregated for analysis. Information related to your identity (such as gender, age, marital status, academic title, etc.) and opinions will only be disclosed after statistical processing (e.g., "Faculty members from 'Double First-Class' universities exhibit the following characteristics").  **Agreement to Collect Personal Data (Tick the corresponding box below)**  □ I agree to provide my personal information as it is necessary for the research purpose.  □ I decline to participate in the research because I do not agree to the collection of my personal information.  **Access to Research Information and Feedback**  -You may request a copy of this Informed Consent Form;  -If there are significant changes to the research protocol, we will provide updated information and re-obtain your consent;  -This research will not provide individual analysis results. However, if you wish to learn about the overall research conclusions, you may contact the principal investigator to request a summary report after the research is completed.  **Compensation for Damages**  If you suffer actual physical or mental harm due to this research, you are entitled to free medical treatment and legal compensation.  **Further Use of Data**  If the research progresses smoothly, your anonymized data may be used in other future academic research studies.  Please tick one of the following options regarding the use of your data in other future research:  □ I agree to my data being used in other future studies.  □ I only agree to my data being used by researchers from Huaqiao University.  □ I only agree to my data being used by the researchers of this study.  □ I do not agree. | | | | | |
| **5.Contact Information**  If you have any questions, doubts, or complaints regarding your participation in this research, please contact the principal investigator.  Name：FUMengyun Tel：13606043917 | | | | | |
| I have read and understood all the above information, confirm my voluntary participation in this research, and acknowledge my rights, the data processing methods, and the research risks. I hereby sign this Informed Consent Form. | | | | | |
| Participant's Name:  Participant's Signature:  Date:    Name of the Person Obtaining the Consent Form:    Signature of the Person Obtaining the Consent Form:  Date: | | | | | |
